# Multilocus sequence typing (and phylogenetic analysis) of *Campylobacter jejuni* and *Campylobacter coli* strains isolated from clinical cases in Greece

**DOI:** 10.1186/1756-0500-6-359

**Published:** 2013-09-08

**Authors:** Vasiliki Ioannidou, Anastasios Ioannidis, Emmanouil Magiorkinis, Pantelis Bagos, Chryssoula Nicolaou, Nicolaos Legakis, Stylianos Chatzipanagiotou

**Affiliations:** 1 Department of Biopathology and Clinical Microbiology, Athens Medical School, Aeginition Hospital, Vass. Sophias av. 72-74, Athens 115 28, Greece; 2 Department of Nursing, Faculty of Human Movement and Quality of Life Sciences, University of Peloponnese, Orthias Artemidos & Plataion, Sparta 23100, GR, Greece; 3 Department of Hygiene, Epidemiology and Medical Statistics, Medical School, University of Athens, Mikras Asias av.75, Athens 11527, Goudi, Greece; 4 Department of Computer Science and Biomedical Informatics, University of Central Greece, Papasiopoulou 2-4, Lamia 351 00, Greece

**Keywords:** Campylobacter jejuni, Campylobacter coli, Serotyping, FlaA typing, PFGE, MLST, Phylogenetic analysis

## Abstract

**Background:**

The molecular epidemiology of *C. jejuni* and *C. coli* clinical strains isolated from children with gastroenteritis, was investigated using the multilocus sequence typing method (MLST). This analysis establishes for the first time in Greece and constitutes an important tool for the epidemiological surveillance and control of *Campylobacter* infection in our country.

**Methods:**

The MLST genotypes were compared with those gained by other typing methods (HS-typing, PFGE and *FlaA* typing) and were also phylogenetically analyzed, in order to uncover genetic relationships.

**Results:**

Among 68 *C. jejuni* strains, 41 different MLST-Sequence Types (MLST-STs) were found. Fifty six strains or 34 MLST-STs could be sorted into 15 different MLST-Sequence Type Complexes (MLST-STCs), while twelve strains or seven MLST-STs did not match any of the MLST-STCs of the database. Twenty *C. coli* strains belonged to 14 different MLST-STs. Eleven MLST-STs were classified in the same MLST-STC (828), and three were unclassifiable. There was no significant association between the MLST-STs and the results of the other typing methods.

Phylogenetic analysis revealed that some strains, classified to the species of *C. jejuni*, formed a separate, phylogenetically distinct group. In eight strains some alleles belonging to the taxonomic cluster of *C. jejuni,* were also detected in *C. coli* and vice versa, a phenomenon caused by the genetic mosaic encountered inside the genus *Campylobacter*.

**Conclusions:**

The MLST-ST determination proved to be a very useful tool for the typing as well as the identification of *Campylobacter* on the species level.

## Background

*Campylobacter* is one of the leading causes of human bacterial gastroenteritis worldwide, with a very wide distribution in food animals. The genus *Campylobacter* comprises many species, among which the most commonly isolated in humans are *Campylobacter jejuni* and *C. coli*[1]. Sporadic cases have been associated with consumption of undercooked poultry meat, while small or large outbreaks have been associated with raw milk and contaminated water [2]. Most cases of human campylobacteriosis are self limiting, requiring no medical treatment. Therapeutic intervention is often warranted in children or in severe or long-lasting infections, the latter occurring mainly in immunocompromised individuals [3].

Due to the impact of *Campylobacter* on public health, epidemiological investigations analyzing the clonality of the isolated strains are very important, in order to trace the sources and routes of transmission, to follow up the temporal and geographic distribution of important phenotypic characteristics and to develop effective strategies for the control and prevention of the pathogen spread, especially inside the food chain [4].

Therefore, numerous phenotyping and genotyping methods are available [5]. Although for the choice of the appropriate method, the rapidity, the cost and the ease of implementation are essential, the main point should be above all the ability in providing accurate, reliable and highly reproducible results [6]. One method meeting the latter condition very well is the multilocus sequence typing (MLST), which has already been developed and used for the molecular typing of *Campylobacter* spp., based on the sequence identification of multiple genetic loci, located in seven housekeeping genes: *aspA, glnA, gltA, glyA, tkt, pgm, uncA*[7-9]. It has proved to be a useful tool for discriminating isolates, for the definition of the structures of the various *Campylobacter* populations and for disclosing associations among sequence types or lineages with the isolation sources or specific virulence factors, thus contributing to the identification of outbreaks and to the appropriate public health intervention [10]. Results from various reports using MLST are imported in international databases, which are open to the enrichment by newly discovered MLST-types, providing an on-going updated global standpoint of *Campylobacter* epidemiology.

The present study aimed to investigate for the first time in Greece the molecular epidemiology of *C. jejuni* and *C. coli* clinical strains isolated from children with gastroenteritis, using the MLST. The bacterial strains included in the study were heat- stable serotyped (HS), genotyped by pulsed field gel electrophoresis (PFGE) and by *flagellin A* typing (*flaA* typing). The MLST genotypes were compared with those gained by the other three typing methods (HS-typing, PFGE and *FlaA* typing) and statistically analyzed. In addition the MLST types were phylogenetically identified on the species level and analyzed, in order to uncover genetic relationships [7,11,12].

## Methods

### Bacterial strains

A total of 68 *C. jejuni* and 20 *C. coli* clinical isolates were collected from infected children up to 14 years from 5 general hospitals of the area of Attica. All the strains were isolated from sporadic cases with gastroenteritis. Bacteria were identified by means of conventional bacteriological standard procedures. Stool samples were cultured for three days at 42°C in Campy enrichment Thioglycolate Medium, supplemented with trimethoprim, vancomycin, polymyxin B, cephalothin & ampotericin B [(Thioglycolate Medium w/0.16% Agar), (REMEL Inc. Kansas U.S.A.)]. Liquid cultures were centrifuged, the supernatants were discarded and the sediments were inoculated in Skirrow agar (Brucella Agar of w/5% Sheep Blood, Trimethoprim, Vancomycin, Polymyxin B, REMEL Inc. Kansas U.S.A.) and incubated for 48 h at 42°C, in a microaerophilic atmosphere for Campylobacter. Identification of typical colony was performed by hippurate hydrolysis and a commercially available identification kit (Api Campy, bioMerieux). Each strain was numerically designated with an internal number, corresponding to the respective clinical specimen sent to the laboratory. Ethical approval was not required under Greek research regulations and all samples were taken as part of standard patient care.

### Heat-stable serotyping

Serotyping was performed as reported previously [13] with a commercially available set of antisera (*Campylobacter* Antisera, “Seiken Set”, Denka Seiken, Japan) based on Penner’s heat-stable (HS) serogroups, and containing 25 absorbed single or group antisera against the most common HS-serotypes. HS-antigen extracts were prepared by the original Penner boiling-method.

### Pulsed-field gel electrophoresis

PFGE was performed as reported previously [14].

### Flagellin gene typing

*FlaA* typing was performed, modified as described by Nachamkin et al. [15,16] using restriction endonuclease *DdeI* (US Biological, Swampscott MA, U.S.A.).

### Bacterial DNA isolation for MLST

Template DNA for the MLST was extracted with the commercially available extraction reagent “instagene matrix” (Bio Rad Laboratories, California, U.S.A.), according to the manufacturer’s instructions.

### Multilocus sequence typing

For the PCR, the commercial ready mix “Jumpstart Red Taq” (SIGMA Laboratories, St. Louis, U.S.A.) was used. The final mix contained 25 μl Jumpstart Red Taq, 1 μM of each primer, 18 μl distilled water and 5 μl template. Primers were provided by Sigma Genosys St. Louis USA in lyophilized form. For each PCR reaction for the detection of each of the seven housekeeping genes (*aspA, glnA, gltA, glyA, tkt, pgm, uncA*) the primer concentration was 10 pmol/μl. The composition of the primer pairs used for the initial PCR for the detection of the housekeeping genes, as well as for the subsequent PCR for the sequencing in order to determine the alleles, has been described previously elsewhere [7,9].

An aliquot of initial PCR sample was analyzed by conventional 1.2% agarose gel electrophoresis. DNA was stained with ethidium bromide and visualized and photographed with a computerized transluminator system (Chemidoc, Bio Rad Laboratories, California, U.S.A.).

### Sequence analysis

The sequencing of the PCR products was performed by Macrogen Incorporation (908 World Merdian Center #60-24 Gasan-dong, Geumchum-gu Seoul, Republic of Korea).

### Statistical analysis

Due to the existence of many independent variables (serotypes and genotypes) and a relative small number of observations, the penalized logistic regression with the LASSO-Least Absolute Shrinkage and Selection Estimator was used.

### Phylogenetic analysis

The electrophoretic patterns gained by the PFGE were expressed with a binary system and symbolized as follows: “A” for the presence of a band and “T” for the absence. The distances between the nucleotide clusters were measured after the model of Jukes and Cantor and the dendrograms (phylogenetic trees) were constructed with the UPGMA (Unweighted Pair Group Method with Arithmetic Mean) method.

For the MLST results, the alignment of all the gene sequences and loci was performed with the algorithm Clustal W using the MEGA (Molecular Evolutionary Genetics Analysis) 5.05 software. The genetic distances between the sequences were calculated using the Kimura 2-parameter model [17]. The phylogenetic trees were constructed using the neighbor – joining method and their reliability was checked by bootstrapping analysis (1000 replicates). One cluster has considered as significant, if it was present in over than 75% of the permuted trees.

## Results

### Serotyping

For *C. jejuni* 22 out of the 68 strains were not typed, while 20 different serotypes or serotype complexes were found in the 46 serotyped strains. From the 20 *C. coli* strains 12 were not typed and 7 different serotypes were detected in the 8 serotyped strains. Some strains gave positive agglutination reactions with more than one antisera (Tables 1 and 2). There was no significant association of a certain serotype with a certain MLST type for both *Campylobacter* species. All the serotypes of *C. coli* were shared also by *C. jejuni*, while the serotype complex HS 37 + HS 57 was found only in *C. coli*.

**Table 1:** **Typing of 68 *C. jejuni *strains by MLST, fla *A *, PFGE and serotyping**

***C. jejuni *strain Nr.**	**MLST-ST**	**MLST-STC**	**Serotype**	***flaA GR *type**	**PFGE**
6	572	206	NT	19	5
10	5213	353	HS 5	20	6
11	354	354	NT	7	7
14	52	52	HS 5	20	8
18	49	49	NT	9	10
23	1723	354	HS 11	39	11
24	50	21	NT	7	4
26	50	21	NT	27	4
34	353	353	HS 3	3	13
38	305	574	HS 5	20	6
40	206	206	NT	19	14
46	61	61	HS 4,13,16,43,50	34	15
47	42	42	NT	30	16
51	61	61	HS 2	12	15
52	3133	22	HS 19 + HS 55	34	17
53	1713	21	HS 2	28	18
67	22	22	HS 23,36,53	19	5
71	443	443	HS 31	19	6
84	50	21	HS 15	23	4
94	38	48	HS 2	33	20
105	3503	48	HS 2	12	22
106	824	257	HS 2 + HS 31	12	23
110	353	353	NT	19	25
132	5218	443	HS 2	1	6
139	45	45	HS 1,44 + HS 12	34	37
142	3504	446	NT	7	27
143	3402		NT	37	10
144	50	21	NT	27	36
153	3505		NT	38	2
154	353	353	NT	1	13
159	3506		NT	7	39
171	52	52	HS 5	20	8
172	2133		HS 15	18	40
175	50	21	HS 8	27	4
176	61	61	HS 4,13,16,43,50	34	15
178	572	206	HS 4,13,16,43,50 + HS 52	19	26
182	2131		HS 15	23	4
185	137	45	NT	31	29
200	2131		HS 15	23	4
201	2131		HS 15	23	4
202	572	206	HS 4,13,16,43,50	19	26
203	572	206	HS 4,13,16,43,50	1	41
210	2133		HS 52	18	40
213	1071		HS 3 + HS 52	6	14
214	5217		HS 52	10	17
216	572	206	HS 4,13,16,43,50	10	14
217	883	21	HS 1,44	18	18
218	50	21	HS 1,44	14	4
219	3505		HS 23,36,53	32	2
227	772	443	HS 37	29	6
228	1474	353	NT	2	42
238	584	257	NT	39	30
240	572	206	NT	19	26
269	572	206	NT	17	26
277	3507	21	HS 52	1	2
278	353	353	NT	1	13
280	1943	21	HS 38	19	24
283	524	353	NT	25	25
295	3503	48	HS 23,36,53	21	8
298	2131		HS 31	24	27
305	42	42	NT	30	31
314	305	574	HS 5	20	32
318	572	206	HS 4,13,16,43,50	10	33
338	1966	22	HS 19	38	17
339	137	45	HS 21	31	35
366	2100	52	HS 5	20	3
369	122	206	HS 4,13,16,43,50	17	43
372	21	21	HS 2	17	4

*ST* Sequence type, *STC* Sequence type complex. Detailed description of the data is given in the main text.

**Table 2:** **Typing of a total of 20 *C. coli *strains by MLST, *flaA*, PFGE and serotyping**

***C. coli *strain Nr.**	**MLST-ST**	**MLST-STC**	**Serotype**	***flaA GR *type**	**PFGE**
13	5214		NT	8	9
15	2073	828	NT	7	9
25	2003	828	HS 1,44	27	12
48	5219	828	HS 15	23	12
56	2003	828	NT	5	19
72	5216	828	NT	8	12
74	2003	828	NT	8	12
102	1582		NT	15	21
147	1595	828	NT	5	38
156	57	828	NT	35	28
162	2503	828	HS 37	8	9
177	2003	828	HS 37 + HS 57	39	34
189	1441	828	NT	10	9
193	1414	828	HS 3	8	28
196	2003	828	HS 31	8	34
211	1595	828	NT	10	9
220	1667	828	HS 38	8	9
239	1671	828	NT	15	9
337	2003	828	HS 31	8	34
368	1605		NT	11	34

*ST* Sequence type, *STC* Sequence type complex. Detailed description of the data is given in the main text.

### Flagellin gene typing

Among the 68 *C. jejuni* and the 20 *C. coli* strains, 28 and 10 different *flaA* types, were detected respectively (Tables 1 and 2). The two species bore five *flaA* types in common: 7, 10, 23, 27 and 39. There was no significant association between a certain flaA type and a certain MLST type.

### Pulsed-field gel electrophoresis

PFGE revealed 35 different genotypes for *C. jejuni* and 7 for *C. coli* (Tables 1 and 2). There was no common genotype among the two species populations.

### Multilocus sequence typing

All the strains were designated to a certain MLST sequence type (MLST-ST) by the combination of the seven allelic housekeeping genes. Moreover, some of them could be classified to a certain MLST sequence type complex (MLST-STC), which represented a clonal complex of a specific combination of four or more allelic genes [9]. Each MLST-ST and MLST-STC is marked with a unique number and the corresponding gene combinations are registered and can be accessed in an international electronic database PubMLST (http://pubmlst.org/campylobacter/).

The MLST-STs and MLST-STCs of the investigated *Campylobacter* strains are depicted in detail in Tables 1 and 2.

Among the 68 *C. jejuni* strains, 41 different MLST-STs were found. Fifty six strains or 34 MLST-STs could be sorted into 15 different MLST-STCs, while the remaining 12 strains or seven MLST-STs did not match any of the MLST-STCs of the MLST database.

The 20 *C. coli* strains belonged to 14 different MLST-STs. Eleven MLST-STs were classified in the same MLST-STC-828 were is common as described previously [18], while the remaining three could not match any MLST-STC.

The numbers of different alleles for each housekeeping gene found in *C. jejuni* were as follows: 13 for *aspA*, 14 for *glnA*, 10 for *gltA*, 10 for *glyA*, 16 for *pgm*, 14 for *tkt* and 9 for *uncA*. For *C. coli* the corresponding numbers were: 1 for *aspA*, 3 for *glnA*, 4 for *gltA*, 5 for *glyA*, 6 for *pgm*, 8 for *tkt* and 5 for *uncA*. The distribution of *C. jejuni* and *C. coli* MLST- STs and STCs from Greece, among strains isolated from various countries (data available on PubMLST) is presented in Tables 3 and 4. There was no significant correlation between the PFGE and the MLST profiles of both *Campylobacter* species.

**Table 3:** The distribution of *C. jejuni *MLST- STs and STCs from Greece among various countries; + stays for presence, - for absence, +* for presence reported for the first time

**MLST- ST**	**MLST- STC**	**GR**	**DK**	**UK**	**NL**	**AU**	**AN**	**US**	**CA**	**ES**	**NO**	**DE**	**LU**	**JP**	**UY**	**CH**	**ZA**	**BE**	**TH**	**IT**	**FR**	**FI**	**SE**	**CZ**
21	21	+	+	+	+	+	+	+	+	+	+	+	+	+	+	+	-	-	-	-	-	-	-	-
22	22	+	+	+	+	+	+	+	+	+	-	+	-	+	+	-	+	+	+	+	-	-	-	-
38	48	+	-	+	+	-	-	+	+	-	-	+	-	+	-	-	-	-	-	-	-	-	-	-
42	42	+	-	+	+	+	-	+	+	-	-	+	-	+	-	-	-	+	-	-	-	-	-	-
45	45	+	+	+	+	+	+	+	+	+	-	+	-	+	-	-	-	+	-	-	+	+	+	-
49	49	+	+	+	+	-	-	-	-	-	-	+	-	-	+	-	-	-	+	-	+	-	-	-
50	21	+	+	+	+	+	+	+	+	+	-	+	-	+	-	+	-	+	+	+	-	-	-	-
52	52	+	-	+	+	+	+	-	+	-	-	-	-	+	+	+	-	-	-	-	-	-	-	-
61	61	+	-	+	+	+	-	+	+	-	-	+	-	+	-	+	-	-	-	-	-	-	-	-
122	206	+	-	+	+	-	-	+	-	-	-	+	-	-	-	+	-	-	-	-	-	-	-	-
137	45	+	-	+	+	-	+	+	+	-	-	+	-	-	-	-	-	+	+	-	-	-	+	-
206	206	+	-	+	+	-	-	-	-	-	-	+	+	-	-	-	-	-	-	-	-	-	-	-
305	574	+	-	+	+	-	-	-	-	+	-	-	-	+	-	-	-	-	+	-	-	-	-	-
353	353	+	-	+	+	-	+	+	+	-	-	+	-	-	-	+	-	-	-	-	-	-	-	-
354	354	+	-	+	+	+	-	+	+	-	-	+	+	+	-	-	-	+	+	-	-	-	-	-
443	443	+	-	+	+	-	-	-	+	-	-	+	-	-	+	-	-	+	-	-	-	-	-	+
524	353	+	-	-	+	+	-	-	-	-	-	-	-	-	-	-	-	-	-	-	-	-	-	-
572	206	+	-	+	+	-	-	-	-	+	-	+	+	-	-	+	-	+	-	-	-	-	-	-
584	257	+	-	+	+	-	-	-	-	-	-	+	-	-	-	-	-	-	-	-	-	-	-	-
772	443	+	-	+	+	-	-	-	-	-	-	-	-	-	-	-	-	-	-	-	-	-	-	-
824	257	+	-	+	+	-	-	-	-	+	-	+	+	-	-	+	-	-	-	-	-	-	-	-
883	21	+	-	+	+	+	-	-	-	+	-	+	+	-	-	-	-	+	-	-	-	-	-	-
1071		+	-	+	+	-	-	-	-	-	-	-	-	-	-	-	-	-	-	-	-	-	-	-
1474	353	+	-	-	-	-	-	-	-	-	-	-	-	-	-	-	+	-	-	-	-	-	-	-
1713	21	+	-	+	-	-	-	-	-	-	-	-	-	-	-	-	-	-	-	-	-	-	-	-
1723	354	+	-	+	-	-	-	-	-	-	-	-	-	-	-	-	-	-	-	-	-	-	-	-
1943	21	+	-	+	+	-	-	-	-	-	-	-	-	-	-	-	-	-	-	-	-	+	-	-
1966	22	+	-	-	-	-	-	-	-	-	-	-	-	-	-	-	-	-	-	-	-	+	-	-
2100	52	+	-	+	+	-	-	-	-	-	-	-	-	-	-	-	-	-	-	-	-	-	-	-
2131		+	-	+	+	-	-	-	-	-	-	-	-	-	-	-	-	-	-	-	-	-	-	-
2133		+	-	+	+	-	-	-	-	-	-	-	-	-	-	-	-	-	-	-	-	-	-	-
3133	22	+	-	+	-	-	-	-	-	-	-	-	-	-	-	-	-	-	-	-	-	-	-	-
3402		+	-	+	-	-	-	-	-	-	-	-	-	-	-	-	-	-	-	-	-	-	-	-
3503	48	+*	-	-	-	-	-	-	-	-	-	-	-	-	-	-	-	-	-	-	-	-	-	-
3504	446	+*	-	-	-	-	-	-	-	-	-	-	-	-	-	-	-	-	-	-	-	-	-	-
3505		+*	-	-	-	-	-	-	-	-	-	-	-	-	-	-	-	-	-	-	-	-	-	-
3506		+*	-	-	-	-	-	-	-	-	-	-	-	-	-	-	-	-	-	-	-	-	-	-
3507	21	+*	-	-	-	-	-	-	-	-	-	-	-	-	-	-	-	-	-	-	-	-	-	-
5213	353	+	-	-	-	-	-	-	-	-	-	-	-	-	-	-	-	-	+	-	-	-	-	-
5217		+*	-	-	-	-	-	-	-	-	-	-	-	-	-	-	-	-	-	-	-	-	-	-
5218	443	+*	-	-	-	-	-	-	-	-	-	-	-	-	-	-	-	-	-	-	-	-	-	-

Abbreviations: *AU* Australia, *BE* Belgium, *CA* Canada, *AN* Curacao, *CZ* Czech Republic, *DK* Denmark, *FI* Finland, *FR* France, *DE* Germany, *GR* Greece, *IT* Italy, *JP* Japan, *LU* Luxembourg, *NL* Nederland, *NO* Norway, *ZA* South Africa, *ES* Spain, *SE* Sweden, *CH* Switzerland, *TH* Thailand, *UK* United Kingdom, *UY* Uruguay, *US* USA*.*

**Table 4:** **The distribution of *C.*** coli MLST- STs and STCs from Greece among various countries; + stays for presence, - for absence, +* for presence reported for the first time

**MLST- ST**	**MLST- STC**	**GR**	**US**	**DK**	**ES**	**LU**	**UK**	**DE**	**SN**	**CA**
57	828	+	-	-	-	-	-	-	-	-
1414	828	+	+	-	-	-	-	-	-	-
1441	828	+	+	-	-	-	-	-	-	-
1582		+	-	+	+	+	-	-	-	-
1595	828	+	-	+	+	+	+	+	-	-
1605		+	-	-	-	-	-	-	+	-
1667	828	+	+	-	-	-	-	-	-	-
1671	828	+	+	-	-	-	-	-	-	-
2003	828	+	-	-	-	-	+	-	-	-
2073	828	+	-	-	-	-	+	-	-	-
2503	828	+	-	-	-	-	-	-	-	+
5214		+*	-	-	-	-	-	-	-	-
5216	828	+*	-	-	-	-	-	-	-	-
5219	828	+*	-	-	-	-	-	-	-	-

Abbreviations: *CA* Canada, *DK* Denmark, *DE* Germany, *GR* Greece, *LU* Luxembourg, *SN* Senegal, *ES* Spain, *UK* United Kingdom, *US* USA.

It must be pointed out, that there was a complete agreement of the MLST-STs of the database, with the conventionally performed identification of the strains on the species level (*C. jejuni* or *C. coli*).

### Phylogenetic analysis

**Figure 1:**
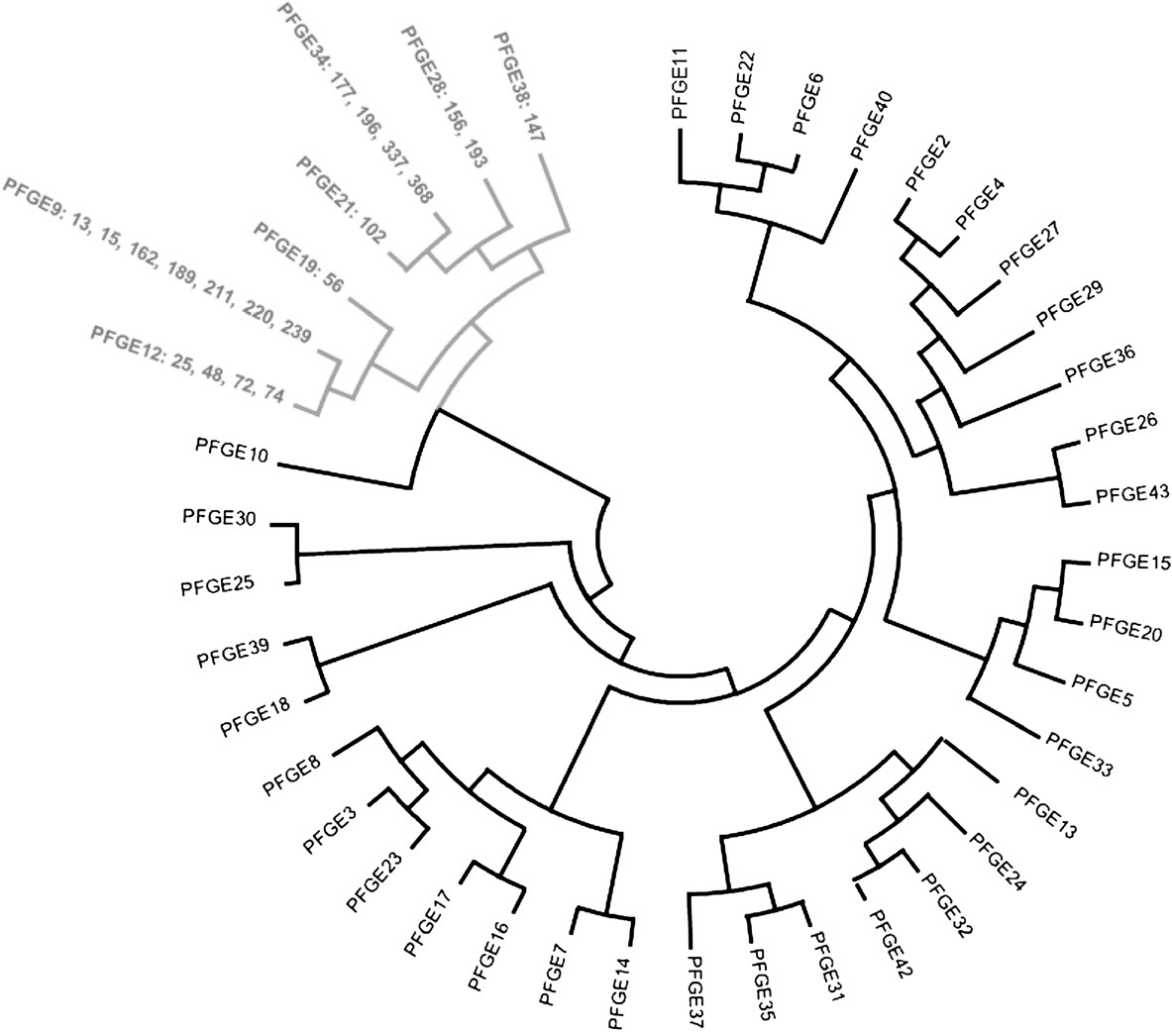
**Phylogenetic tree composed by the PFGE patterns: in black the *C. jejuni *cluster and in gray the *C. coli *cluster.**

**Figure 2:**
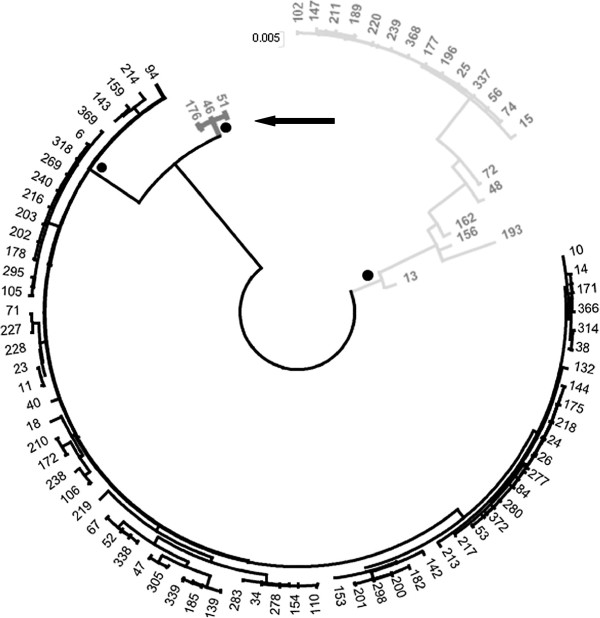
**Phylogenetic tree composed by means of the seven housekeeping genes of the MLST analysis: in black the *C. jejuni *cluster and in gray the *C. coli *cluster.** The dots show the confidence intervals (bootstrapping) >75%. The distinct *C. jejuni* cluster of MLST-ST 61 is marked by the arrow.

**Figure 3:**
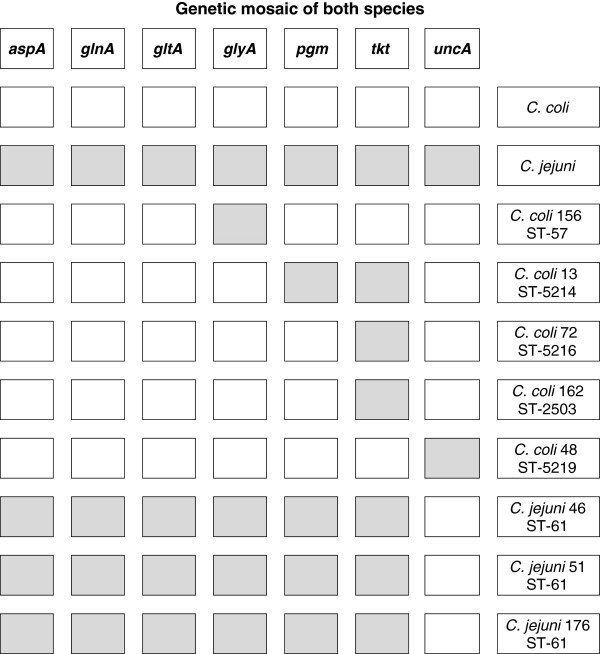
**The genetic mosaic detected in eight strains of both species, bearing alleles belonging taxonomically to the other species cluster.** In gray the *C. jejuni* alleles and in white the *C. coli* alleles.

Phylogenetic analysis aimed to classify the various genotypes by constructing phylogenetic trees based on the patterns gained by PFGE and MLST (Figures 1 and 2). PFGE results are representative for the entire genome, but the method is biased by selective forces, while the MLST patterns are suitable for phylogenesis and global epidemiology as they derive from the combination of the allelic forms of the seven housekeeping genes. For that purpose the electrophoretic patterns were converted to binary numbers. The results arising from both analytical methods were in complete agreement with each other and the strains were grouped together by species (*C.jejuni* and *C. coli*) in two distinct clusters. Phylogenetic analysis of the MLST patterns revealed that some strains, although classified to the species of *C. jejuni*, they formed a separate, phylogenetically distinct group, a point of epidemiological interest (Figure 1). The paradox arising from the MLST phylogenetic patterns, was that in eight strains some alleles belonging to the taxonomic cluster of *C. jejuni,* were also detected in *C. coli* and vice versa, a phenomenon already described in previous reports [7,11,12], caused by the genetic mosaic encountered inside the genus *Campylobacter*. The genetic mosaic of these eight particular strains is shown in Figure 3. Regarding the number of mosaic alleles, for *C. jejuni* there was only one allele recorded in the *uncA* locus and was specific for ST-61, and for *C. coli,* there were three alleles in the *tkt* locus and one in each of the *uncA, pgm* and *gltA* loci. The distribution of mosaic genes was asymmetrical between *C. jejuni* and *C. coli,* and only one of the mosaic genes showed horizontal transfer from *C. coli* to *C. jejuni* STs, while four mosaic genes from *C. jejuni* into *C. coli*[12].

## Discussion

The results confirm the high discriminatory power of the MLST analysis for *Campylobacter* genotyping, which is applicable worldwide [6,19-21]. The advantage of MLST over the other described genotyping techniques arises from its standardization by the use of seven housekeeping genes and the subsequent sequence analysis of the products, warranting very reproducible and reliable results. Moreover the MLST-ST determination proved to be a very useful tool for the identification of *Campylobacter* on the species level. This is not applicable regarding the MLST-STC determination. *C. jejuni* and *C. coli* share approximately 86.5% identity at the nucleotide sequence level within the MLST loci [7]. Therefore strains of these two species can belong to the same MLST-STC but not to the same MLST-ST. The comparison of results obtained by other genotyping techniques is often difficult, due to the diversity of the used performance protocols among the various laboratories. In the present study the MLST analysis yielded more genotypes than the other two methods (PFGE and *flaA* typing).

Among the 88 *Campylobacter* strains included in the study, 55 different MLST genotypes were found and some of them are reported for the first time (Tables 3 and 4). The absence of prevalence of a particular genotype is consistent with the aspect, that there is considerable diversity among the *Campylobacter* strains isolated in the area of Attica [2]. As far as we know, there is no report from Greece about campylobacteriosis outbreaks during the last thirty years, all the clinical cases being sporadic [2,13,14,22]. This observation is enforced in our investigation by the phenotypic and genotypic diversity found by the other typing methods, as well by the absence of association of any specific phenotype or genotype (serotype, PFGE or *flaA*) with a certain MLST type. In addition, the phylogenetic analysis provided a very fine discrimination with respect to the identification and the clonal distribution of the strains, revealing also mosaic genotypes derived through interspecies recombination of the housekeeping genes between *C. jejuni* and *C. coli* strains. These findings represent an asymmetric gene flow, (4.69% in *C. jejuni* vs 25% in *C. coli*)*,* also described elsewhere [11,12].

## Conclusions

The MLST typing as well as the phylogenetic analysis of *Campylobacter* clinical strains isolated from Greece is presented for the first time in the present study and significantly completes the epidemiological database kept in our laboratory. This database includes, besides the already described phenotypic and genotypic features, all the demographic and clinical data of the patients and constitutes an important tool for the epidemiological surveillance and control of *Campylobacter* infection in our country.

## Abbreviations

FlaA typing: *Flagellin A* typing; HS: Heat- stable; MEGA: Molecular evolutionary genetics analysis; MLST: Multilocus sequence typing; MLST-ST: Multilocus sequence typing-sequence type; MLST-STC: Multilocus sequence typing-sequence type complex; UPGMA: Unweighted pair group method with arithmetic mean.

## Competing interests

The authors declared that they have no competing interests.

## Authors’ contributions

VI performed the experimental part, AI and EM worked out the methodology, PB performed the statistical analysis, CN, NL and SC designed the study, and SC wrote the manuscript. All authors read and approved the final manuscript.
